# Arginine deiminase expressed *in vivo*, driven by human telomerase reverse transcriptase promoter, displays high hepatoma targeting and oncolytic efficiency

**DOI:** 10.18632/oncotarget.17032

**Published:** 2017-04-11

**Authors:** Hui Jiang, Song Guo, Dan Xiao, Xuzhao Bian, Jie Wang, Ying Wang, Huiting Zhou, Jun Cai, Zhongliang Zheng

**Affiliations:** ^1^ State Key Laboratory of Virology, College of Life Sciences, Wuhan University, Wuhan 430072, China; ^2^ Department of Orthopedic, Wuhan Puai Hospital, Wuhan 430034, China; ^3^ Department of Gastroenterology, Jianghan University Affiliated Hospital, Wuhan 430000, China; ^4^ Hubei Collaborative Innovation Center for Industrial Fermentation, College of Biotechnology, Hubei University of Technology, Wuhan 430068, China

**Keywords:** arginine deiminase, ADI, arginine starvation, cancer-targeting therapy, hTERT promoter

## Abstract

Arginine starvation has the potential to selectively treat both primary tumor and (micro) metastatic tissue with very low side effects. Arginine deiminase (ADI; EC 3.5.3.6), an arginine-degrading enzyme, has been studied as a potential anti-tumor drug for the treatment of arginine-auxotrophic tumors. Though ADI-PEG20 (pegylated ADI by PEG 20,000) already passed the phase I/II clinical trials [1], it is just used as adjuvant therapy because of its low efficiency and less targeting. Then, this paper discussed the efficiency of arginine starvation mediated by ADI expressed in cytoplasm for liver cancers. In order to guarantee the tumor targeting, human telomerase reverse transcriptase (hTERT) promoter was used to drive the expression of ADI *in vivo*. To access the anti-tumor efficiency of ADI, p53 gene was used as the positive control. Thus, ADI displayed obvious cytotoxicity to BEL7402 and HUH7 cell lines in cytoplasm. The apoptosis rates rose from 15% to nearly 60% after changing the expression vectors from pcDNA4 plasmid to adenovirus. Compared with p53-adenovirus, ADI-adenovirus showed the higher oncolytic activity in the intratumoral injection model of mice. Tumor disappeared after the treatment of ADI-adenovirus for two weeks, and the mice pulled through all. Therefore, ADI is an ideal anti-tumor gene for caner targeting therapy with the help of hTERT promoter.

## INTRODUCTION

Cancer is the most common cause of death in the world, which is a group of diseases involving uncontrolled growth and spread of abnormal cells. Cancer gene therapy is the method to introduce therapeutic genes into cancer cells for the treatment of cancer. As a consequence, there have been over 1800 clinical trials of gene therapy already conducted or currently ongoing worldwide [2] with an increased understanding of the genetic basic of many diseases, improvement of vectors to minimize unwanted toxicity, advances in approaches for manipulating DNA expression and delivery in a target-specific manner [3]. Cancer has already become one of the major disease targets for gene therapy over the past decades. However, there are still some impediments to intervene cancer therapeutic effects, such as correct choice of optimal cancer-suppressing gene, precise utility of efficacious doses, potential toxicity from the overexpress of endogenous therapeutic gene, and so on.

ADI is a potential cancer therapy agent for the treatment of arginine-auxotrophic tumors, such as hepatocellular carcinomas and melanomas [4]. ADI originally derived from Mycoplasma bacteria, has high immunogenicity that could limit its clinical utility. But Phase I trial indicated that ADI-PEG20 just had reasonable toxicity in combination with docetaxel [5]. So ADI-PEG20 is used as an effective anticancer medicine. However, ADI needs 4 days to make tumor cells apoptosis [6]. Compared with tumor targeting medicines, its tumor-suppressing efficiency is a little bit lower.

Our group adopted a new strategy to improve the tumor-suppressing efficiency of ADI. ADI was expressed directly in tumor cells to exhaust endogenous and exogenous arginine. As an exogenous protein, the enzyme activity of ADI expressed in cytoplasm will not be interrupted theoretically by any other intracellular factors through interaction or competitive inhibition. Hence, the tumor-suppressing efficiency of ADI will be improved quickly and directly. Compared with other gene delivery systems, adenovirus was chosen as the gene-transfer vector because of its virtues [7]. In order to enhance the tumor-targeting ability of ADI, hTERT promoter was used to replace the CMV promoter in adenovirus vector and drive the expression of ADI in cancer cells. In this paper, we discussed the activity of hTERT promoter in the different cancer cells. What's more, we investigated the tumor-suppressing efficiency of ADI expressed in cytoplasm as an exogenous gene.

## RESULTS

### The activity assay of the endogenous hTERT promoter in the different cell lines

**Figure 1:**
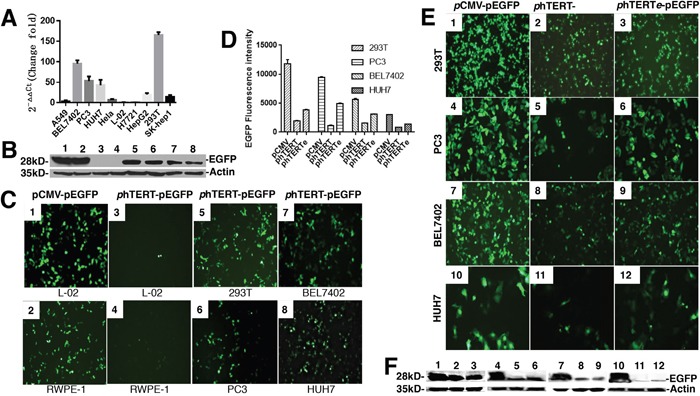
The activity assay of phTERT promoter in different cell lines **(A)** Comparison of the expression of endogenous hTERT protein in different cell lines by real-time PCR. GAPDH was the internal reference. **(B)** Immunoblots of GFP expression driven by phTERT promoter in different cell lines. The data were according to Figure 1C. C-myc-tag antibody was used to detect c-myc-tag-fused ADI and p53. **(C)** The activity detection of exogenous phTERT promoter in different cell lines by the transient transfection of phTERT-pEGFP vector. pEGFP-N1 vector was used as a positive control. The fluorescence of EGFP protein was detected by OLIMPUS inverted fluorescence microscope SteREO Discovery V12. **(D)** The quantification analysis of GFP fluorescence by Image-J 1.5. The data were according to Figure 1E. **(E)** Activity comparison of three promoters which were pCMV, phTERT and phTERTe by checking GFP fluorescence. **(F)** Immunoblots of GFP expression in 293T, PC3, BEL7402, and HUH7 cells according to Figure 1D. C-myc-tag antibody was used to detect c-myc-tag-fused ADI and p53.

The activity of phTERT promoter was verified in different cell lines. The cell lines were chosen according to the results from the luciferase report gene driven by phTERT promoter [8–10]. Some cell lines possessing the higher activity of endogenous hTERT promoter were selected for further tests. The expression levels of endogenous hTERT protein were detected by fluorescence quantitative real-time PCR in different cell lines. The mRNA of hTERT gene was used as PCR target. As shown in Figure 1A, the mRNA levels of hTERT gene were lower in the cell-lines of A549, Hela, L-02, H7721, HepG2 and SK-hep1, which indicated that hTERT protein was not expressed in all of the cancer cells. But several cell-lines still possess the higher level of hTERT obviously as shown in Figure 1A, such as 293T, BEL7402, PC3, HUH7. Therefore, these four cell-lines were used to test the activity of exogenous phTERT promoter.

GFP gene is an ideal report gene for testing the activity of exogenous promoters. The vector of pEGFP-N1 was chosen for the tests. pCMV promoter was replaced by phTERT promoter in the pEGFP-N1vector. The restriction enzymes, Ase I and Bgl II, were used as the cutting sites. New constructed plasmid, phTERT-pEGFP-N1, was transfected into endogenous phTERT-positive cell-lines. Then we checked the fluorescence of the cell-lines on an inverted fluorescence microscope after 48h cell-cultured time. As shown in Figure 1C, there wasn't the green fluorescence in two normal cell-lines, L-02 and RWPE-1. But other four positive cell-lines emitted the strong green fluorescence. The expression results of EGFP protein in Figure 1B corresponded to the fluorescence results in Figure 1C. There weren't the expressions of EGFP protein in L-02 and RWPE-1 cell lines under the regulation of the phTERT promoter. 293T, PC3, HUH7 and BEL7402 cell lines displayed the obvious expressions of EGFP protein. Compared with pCMV promoter in the pEGFP-N1vector, phTERT promoter has the relative lower transcription efficiency which resulted in the relative weaker fluorescence of the cell lines. These results indicated that the exogenous phTERT promoter didn't present the enough efficiency though it could effectively launch the expression of EGFP protein in the cancer cells maintaining the high activity of endogenous hTERT promoter.

It is an effective way to add a suitable enhancer at the 5’-end of phTERT promoter in order to improve the activity of phTERT promoter. We chose the enhancer of pCMV promoter after investigating several enhancers [11, 12]. phTERTe promoter was synthesized, including the pCMV enhancer and phTERT promoter. phTERTe-pEGFP-N1 plasmid was constructed and transfected into cell-lines. As shown in (Figure 1D, 1E and 1F), phTERTe promoter resulted in the stronger fluorescence and the higher expression of EGFP protein than phTERT promoter, but still was relative weaker than pCMV promoter. In addition to the tumor-targeting specialty, the activity of phTERTe promoter is enough to ensure the moderate expression of cancer suppressor genes in cancer cells. Therefore, phTERTe promoter is an ideal promoter for cancer gene therapy.

### Tumor-targeting effects and cytotoxicity assay of ADI expression plasmid driven by phTERTe

ADI gene is an unexceptionable tumor-targeting gene for cancer gene therapy. To test its cytotoxicity for tumor cells and normal cells, we transferred ADI gene into different cell-lines to induce cell death.

Firstly, pcDNA^TM^4/TO/myc-His vector was used as the transfection vector. Its pCMV promoter was replaced by phTERTe promoter. Restriction enzymes were Mlu I and Afl II. ADI gene and p53 gene were inserted into its multiple cloning sites. Its c-myc tag was kept and fused with the fragments inserted. Cloning details were displayed in Table 1. p53 gene is known as an effective tumor suppressor gene [13], which has been researched for nearly 30 years [14]. p53 gene was used as the positive control.

**Table 1:** The list and sequence of the primers used for plasmid construction

NO	Plasmid Name	Insert Fragment	base number	Restriction Enzyme	Primers
1	phTERT-pEGFP-N1	phTERT	294bp	Ase I	*phTERT_AseI_f*: GATAT ATTAAT GTCTGGATTCCTGGGAAGTCC
				Bgl II	*phTERT_BglII_r*: GATAT AGATCT CTTCCCACGTGCGCAGCA
2	phTERTe-pEGFP	pCMVenhancer-phTERT	595bp	Ase I & Bgl II	synthesised sequence including pCMV's enhancer, phTERT, and restriction enzyme.
3	phTERTe-pcDNA4	pCMVenhancer-phTERT	595bp	Mlu I	phTERT*_MluI_f: gATAT ACGCGT CGTTACATAACTTACGGTAAATGGCCCG
				Afl II	phTERT*_AflII_r: gATAT CTTAAG GGCCAGGGCTTCCCACGTG
4	phTERTe-pcDNA4-p53-cmyc	p53	1200bp	BamH I	*p53_BamHI_F*: GATAT GGATCC GCCATGGAGGAGCCGCAGTC
				Xho I	*p53_XhoI_R*: GATAT CTCgAg GTCTGAGTCAGGCCCTTCTGT
5	phTERTe-pcDNA4-ADI-cmyc	ADI	1233bp	EcoR I	*ADI_EcoRI_f*: gATAT GAATTC ACC ATGTCCGTCTTCGATAGCAAGTTC
				Xho I	*ADI_XhoI_r*: gATAT CTCgAg CCATTTGACATCTTTTCTGGACAGTG
6	phTERTe-Ad5	pCMVenhancer-phTERT	595bp	Sbf I	phTERT*_SbfI_f: GATAT CCTGCAGG CGTTACATAACTTACGGTAAATGGCCCG
				Kpn I	phTERT*_KpnI_r: GATAT GGTACC GGCCAGGGCTTCCCACGTG
7	phTERTe-Ad5-ADI-myc	ADI-cmyc	1233bp	Kpn I	*ADI_KpnI_F1*: GATAT GGTACC ACCATGTCCGTCTTCGATAGCAAGTTC
				EcoR V	*ADI_EcoRV_R1*: GATAT GATATC TCA CAGATCCTCTTCTGAGATGAGT
8	phTERTe-Ad5-p53-myc	p53-cmyc	1200bp	Kpn I	*p53_KpnI_F1*: GATAT GGTACC GCCATGGAGGAGCCGCAGTC
				EcoR V	*p53_EcoRV_R1*: GATAT GATATC TCA CAGATCCTCTTCTGAGATGAGT

Plasmids were named as the type of promoter(p)-vector-gene-tag.

**Figure 2:**
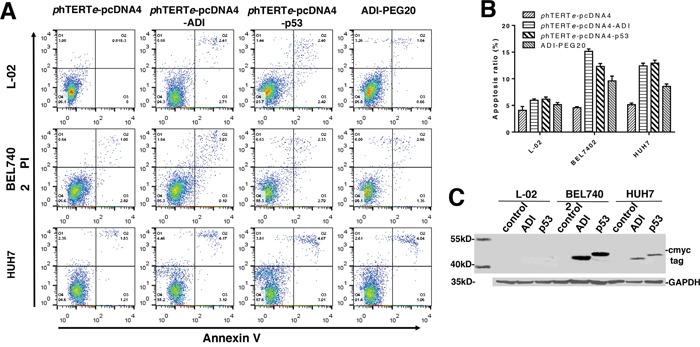
Detecting tumor targeting and cytotoxicity of ADI regulated by phTERTe promoter Cells were transiently transfected by different plasmids including phTERTe-pcDNA4-ADI, phTERTe-pcDNA4-p53 and phTERTe-pcDNA4. Cell apoptosis ratio were checked by flow cytometry after 48h's static cell culture. **(A)** Representative images of FACS analysis of annexin V and PI staining of L-02, BEL7402 and HUH7 cells. **(B)** Summary of FACS analysis from Figure 2A. **(C)** Immunoblots of ADI and p53 expression in L-02, BEL7402 and HUH7 cells. C-myc-tag antibody was used to detect c-myc-tag-fused ADI and p53.

Secondly, having built and verified the hepatic cancer cell model, we just focused on the hepatoma cytotoxicity of ADI. Then new constructed plasmids, phTERTe-pcDNA4-ADI-cmyc and phTERTe-pcDNA4-p53-cmyc, were transfected into L-02, BEL7402 and HUH7 cell-lines. After 48h of cell culture time, cell apoptosis was analyzed by flow cytometer. At the same time, 10 mIU/ml−1 ADI-PEG20 [1, 5] were used as the positive control. As shown in Figure 2A and 2B, BEL7402 cell-line displayed about 15% of apoptosis rate after induced by ADI and p53. HUH7 cell-line got almost 12% of apoptosis rate. L-02 cell line as the negative control only had nearly 6% of apoptosis rate. Thus it can be seen that ADI gene also has the ability to induce the cell apoptosis as p53 gene. By contrast, the apoptosis rate induced by ADI-PEG20 was only about 10% in BEL7402 and HUH7 cell lines, which was lower than the rates aroused by the cytoplasm-expressed ADI. Then, the cytoplasm-expressed ADI possessed the higher cytotoxicity against the hepatoma cells. But due to the different activity of phTERTe promoter in the different cell-lines, BEL7402 cell-line had the highest expression level of ADI and p53 protein (as shown in Figure 2C) which directly led to its highest apoptosis rate.

Based on above results, we can see that ADI gene is a significant anticancer gene for cancer gene therapy, which can successfully induce cell death and presented lower cytotoxicity on normal cell-line L-02 as shown in Figure 2A and 2B. The tumor-targeting efficiency of phTERTe promoter is also very excellent. L-02 cell line as the normal cell control didn't almost have cell death. But expression quantity and time were not so ideal. It follows that expression plasmid is not a good expression vector *in vivo* because its expression efficiency will be determined by its transfection efficiency.

### Tumor-targeting effects and cytotoxicity assay of ADI-adenovirus driven by phTERTe promoter

**Figure 3:**
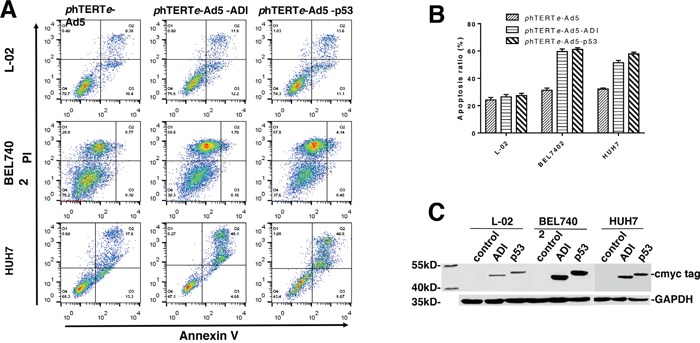
Apoptosis efficiency induced by ADI adenovirus in L-02, BEL7402 and HUH7 cells Cells were separately infected by phTERTe-Ad5, phTERTe-Ad5-ADI and phTERTe-Ad5-p53 adenovirus. Cell apoptosis rate were checked by flow cytometry after the static cell culture for 48h. **(A)** Representative images of FACS analysis of annexin V and PI staining of L-02, BEL7402 and HUH7 cells. **(B)** Summary of FACS analysis from Figure 3A. **(C)** Immunoblots of ADI and p53 expression in L-02, BEL7402 and HUH7 cells. C-myc-tag antibody was used to detect cmyc-tag-fused ADI and p53.

Adenovirus has been developed into very excellent vectors for gene therapy in the past few years. As the fifth generation adenovirus vector, pacAd5/CMV/K-N/pA vector has many advantages, such as easy operation, easy modification, high transfection rate, high expression, and so on. So it was used as our gene therapy vector. Firstly, pCMV promoter was replaced by phTERTe promoter in pacAd5/CMV/K-N/pA vector. Sbf I and Kpn I were used in phTERTe-Ad5 plasmid construction. Secondly, ADI-cmyc gene fragment was amplified from phTERTe-pcDNA4-ADI-cmyc plasmid and was inserted into multiple cloning site of phTERTe-Ad5 plasmid. Then, phTERTe-Ad5-ADI-cmyc plasmid was constructed successfully. phTERTe-Ad5-p53-cmyc was also constructed as the positive control in the same way. Thirdly, the recombinant adenoviruses were packaged out and were used to infect tumor cells. The apoptosis results from flow cytometry were shown in Figure 3A and 3B. ADI-adenovirus was more efficient than ADI plasmid to induce cancer cell death. It was only 48h that death rate raised to nearly 60% in BEL7402 and HUH7 cell-lines. But L-02 cell-line also had approximate 30% of death rate, which implied that ADI-adenovirus is also toxic to normal cells. But the toxicity for normal cells should be resulted from adenovirus itself, not from ADI gene, because there was no big difference of apoptosis rates between ADI/p53-adenovirus-treated L-02 cells and empty adenovirus-treated L-02 cells.

### Oncolytic efficiency of ADI-adenovirus driven by phTERTe promoter

The animal model was constructed by injecting BEL7402 cell-line into the mice. BEL7402 cell-line is an ideal cell-line for our cancer-model based on its high activity of endogenous hTERT promoter. Another reason is that the wild type p53 and myc protein [15, 16] are less expressed in BEL7402 cell-line. So we can use the c-myc tag to detect the expression of ADI and p53 proteins, then compare their oncolytic efficiency in the mice model.

**Figure 4:**
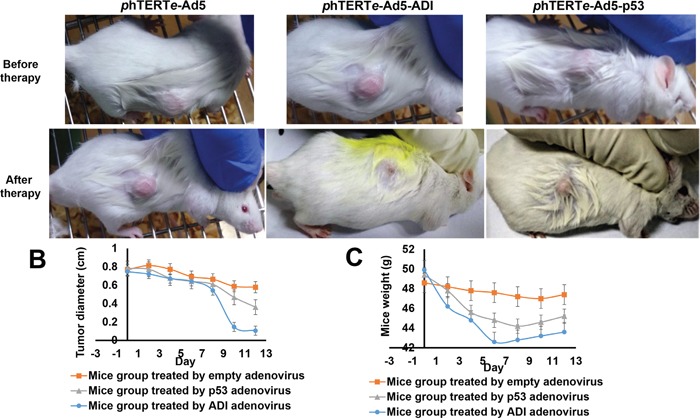
ADI adenovirus driven by phTERTe promoter successfully dissolved the tumors in mice hepatic tumor model **(A)** Compared the therapeutic efficiency of ADI adenovirus and p53 adenovirus after the treatment for 6 days. **(B)** The changes of tumor sizes during gene therapy in mice model. **(C)** The changes of mice weights during gene therapy in mice model.

BEL7402 cells were injected into the right subcutaneous axillary of each mouse to form tumors. When palpable tumors developed into almost 50 mm^3^, recombinant adenoviruses were injected into the tumors. As shown in Figure 4A, Tumors dismissed completely after treated by ADI-adenovirus for two weeks. The tumors treated by p53-adenovirus just decreased the sizes, not disappeared thoroughly. The sizes of negative control tumors were almost the same as the sizes before the injection of empty-adenovirus. What really interested us was that the tumor sizes reduced faster when the mice were treated by ADI-adenovirus for eight days, as shown in Figure 4B. The tumors from other mice reduced their sizes very slowly. The weight of mice also decreased very faster when treated by ADI-adenovirus. But after the injection of ADI-adenovirus for 6 days, the mice weight increased faster than others as shown in Figure 4C.

**Figure 5:**
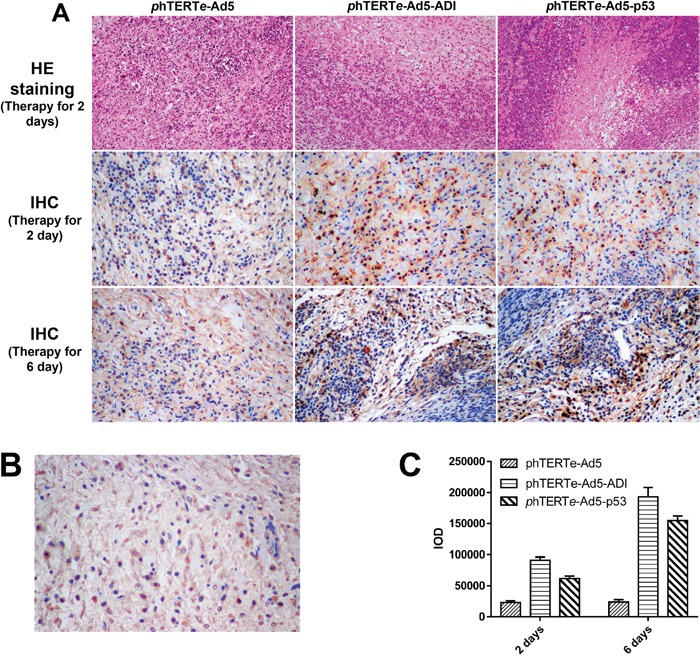
HE and IHC staining of tumor tissues from gene therapy mice **(A)** Detected the expressions of ADI and p53 protein by IHC staining. The tumors treated for 2 days or 6 days were cut out for HE and IHC staining. C-myc-tag antibody was used for IHC staining to detect cmyc-tag-fused ADI or p53 protein. **(B)** Blocking peptide control was used to verify the specificity of c-myc-tag antibody in IHC staining. A synthetic c-myc-tag peptide was used to block the activity of c-myc-tag antibody. **(C)** The expression quantifications of ADI or p53 protein were analyzed by Image-Pro Plus 6.0. IOD (Integrated Option Density) was checked out by calculating the area and the density of the brown color in IHC staining.

Therefore, it can be concluded that ADI-adenovirus has very strong oncolytic activity. The strange thing is that the oncolytic activity of ADI-adenovirus is higher than one of p53-adenovirus, but their cancer cytotoxicity is almost same. Then, we took out the tumors treated for six days to check the expression of exogenous proteins by immunohistochemistry. As the HE-dyed results shown in Figure 5A, the tumor cells treated by ADI- or p53-adenovirus became atrophic and sparse. There were more red-dyed areas between the ADI-treated cells, which suggested that a large amount of cytoplasm was released from dead cells. As the immunohistochemistry results shown in Figure 5A and 5C, the expression of ADI was higher than that of p53. ADI expressed in cytoplasm quickly led to the death of the tumor cells. Large amounts of ADI were released into tumor microenvironments and made the tumors vesiculate inside.

Some pathological phenomenon was observed during the injection of virus, such as spasm, shiver, apocleisis, and so on. These symptoms were very obvious during the initial eight days. The pus was found to exude from the injection position at the surface of the tumors in this period. Then, we always helped to extrude all of the pus before the next injection. The mice started to recover after the treatment for eight days. The mice began to drink and eat, and not to cramp or shiver any more.

## DISCUSSION

Compared with radiotherapy and chemotherapy, cancer gene therapy is accepted by more and more clinicians and patients, which highly become the trend of cancer therapy. The rapidly development of gene therapy arouses a number of innovative treatments for cancer patients. The genetic modification of cancer and immune cells led to numerous successful clinical trials. The use of oncolytic virus and bacteria already obtained several impressive products. But some of the key scientific and regulatory issues still existed in clinical trials, including tumor targeting, safety of transgene vectors, long-term persistence, necessity of integrating into genome, shedding of a virus, immunogenicity of a virus, and so on.

Tumor targeting was the most important factor for gene therapy medicines. Targeted cell immunotherapy will become the future to fight against cancers [17]. Donor nature killer (NK) cell therapy for cancers made very little achievements in clinic due to its poor *in vivo* survival and lack of specificity [18]. Donor cytokine-induced killer (CIK) cell therapy also couldn't eliminate tumors efficiently without combination therapy with chemotherapy in clinic because it had no tumor targeting [19]. However, CAR-T cell is driving the road from laboratory to the clinic [20]. Chimeric antigen receptors (CARs) endow T cell populations with defined antigen specificities that function independently of the natural T cell receptor and permit targeting of T cells towards virtually any tumor [21]. In contrast to donor T cells, CAR-engineered NK cells possess higher cytotoxicity against resistant cancer cells and made more achievements in clinical trials [22]. However, cellular immune therapy still has some scientific and clinical problems to be resolved, such as dangerously high fevers and precipitous drops in blood pressure caused by cytokine-release syndrome, lower activity of immune cells separated from the blood of old patients, short-term persistence leaded by too expensive cellular therapy price and lower cell dosage [23].

In October 2015, the U.S. Food and Drug Administration approved the first oncolytic virus therapy to treat melanoma. The virus used in the treatment is called talimogene laherparepvec (Imlygic), or T-VEC [24]. Oncolytic virus therapy is a new type of immunotherapy that uses genetically modified viruses to only kill cancer cells except healthy cells [25]. The viruses are injected into the tumors and make copies of itself in cancer cells. As a result, the cancer cells burst and die, then release cancer antigens. This triggers the patient's immune system to launch an attack on all cancers cells in the body [26].

We constructed our oncolytic virus in a different way. Our oncolytic virus relies on transferring tumor suppressor gene to kills cancer cells, then makes cancer cells burst and release cancer antigens. Adenovirus vector pacAd5 from Cell Biolabs Inc was chosen as our gene-transferred vector, based on Adenovirus advantages in clinical trials. Adenoviruses are well studied and can be grown into high titer stocks. They can infect different body tissues and be maintained *in vivo* as an episome [27]. More than 400 gene therapy trials have been or are being conducted with human adenovirus vectors [28]. Most of these trials are for treatment of cancers. Nearly all clinical trials have indicated that adenovirus vectors are safe and well tolerated [28].

We applied two strategies to keep the tumor targeting of our oncolytic virus. One was the utility of phTERT promoter, and the other was the utility of ADI gene. hTERT protein has been identified as a major protein involved in aberrant cell proliferation, immortalization, metastasis and stemness maintenance in a majority of tumors, yet it has little or no expression in the normal somatic cells, which makes hTERT promotor become an ideal regulatory element in cancer gene therapy [29]. Over past decades, the therapies based on phTERT promoter such as soft tissue sarcoma [30], leukemia [31], urothelial cancer [32], have developed to be critical and specific for eradicating all types of cancer [33]. Meanwhile, ADI-PEG20 as a potential antineoplastic drug already passed the phase I/II clinical trials [1, 5]. Many tumors need take the arginine from microenvironment due to the deficiency of argininosuccinate synthetase (ASS). ADI protein can use up the arginine in the tumor microenvironment, then leads the tumors to the starvation. But the normal tissues will not be affected because of the presence of ASS in the normal tissue cells [34]. However, ADI protein doesn't possess the enough efficiency to starve tumor cells to die fast, which needs 4 days to make tumor cells apoptosis [6, 35].

In order to improve the anti-tumor efficiency of ADI protein, we tried to express ADI protein in the tumor cells transferred by adenovirus. Compared with p53 gene, ADI gene regulated by phTERT promoter displayed stronger oncolytic activity in the mice model as shown in Figure 4 and 5. So we inferred that, ADI protein expressed *in vivo*, as an exogenous protein, will not theoretically be limited by interaction with other intercellular factors. ADI protein can keep the high efficiency to maintain its tumor-targeting specificity in tumor cells. However, p53 protein as an endogenous protein, has too many interactions with other intercellular proteins *in vivo*, which decreases the anti-tumor activity of p53. Moreover, p53 has no selectivity to induce the apoptosis of cancer cells or normal cells. Therefore, ADI gene is an ideal anti-tumor gene for cancer gene therapy.

We had also tried to delivered ADI-adenovirus by both intravenous and intraperitoneal injection. To our disappointment, the injected mice appeared serious spasm and shiver symptoms both. The injected mice started to die after four days of intravenous or intraperitoneal injection. Obviously, this ADI-adenovirus was not fit for intravenous or intraperitoneal injection at the current stage. There are no tumor ligands on the surface of ADI-adenovirus, which makes ADI-adenovirus unable to target the tumors in blood vessel or peritoneum. On the other side, ADI-adenovirus will arouse serious immunogenicity reaction as other p53-adenovirus in clinics [36]. Therefore, our ADI-adenovirus is more suit for the intratumoral injection in cancer gene therapy.

Therapeutic schemes of cancers will be determined based on a patient's tumor specifics, genetics, host immune status, and so on [37]. Hybrid tumor treatments will make cancer a manageable disease [2]. Our ADI-adenovirus doesn't possess the replicating ability in other tissue cells, which will decrease its side-effects in clinic. Our ADI-adenovirus will be suited to treat the tumors which can't be executed by surgery. Perhaps, a suitable schedule of virus injection will be helpful to melt those tumors in clinic, which still needs be verified by vast quantities of animal experiments in the future. As the research mature of our ADI-adenovirus, it may be used alone or in combination with other current treatments.

Right now, the apoptosis mechanism induced by ADI expressed in the cells is studying in our research group. Next stage, we will describe the molecular details of cell dysfunction aroused by arginine starvation.

## MATERIALS AND METHODS

### Cell culture

Cell lines were cultured in the medium recommended by China Center For Type Culture Collection (CCTCC), including PC3 (from human Caucasian prostate adenocarcinoma), HeLa (from human cervix epithelioid carcinoma), A549 (from human Caucasian lung carcinoma), HepG2 (from human hepatocellular carcinoma), 293T (from human embryonic kidney), BEL7402 (from human hepatocellular carcinoma), H7721 (from human hepatocellular carcinoma), SK-Hep1 (from human endothelial hepatocellular carcinoma) and HUH7 (from human hepatocellular carcinoma). The adenovirus proliferation cell line 293 was grown in DMEM medium supplemented with 10% FBS, 100 IU/ml penicillin, 100 μg/ml streptomycin, and 2 mM L-glutamine. All cells were cultured in a humidified atmosphere containing 5% CO2 at 37°C. All culture reagents were purchased from Life Technologies LLC.

### Plasmid construction and generation of phTERT-Ad5-ADI

The core promoter of hTERT gene was synthesized by Genscript LIC, which contained 284bp from -279 to 5 (numbered from start code ATG). The core promoter of hTERT gene was named as phTERT promoter here. phTERT promoter was inserted into pEGFP-N1 vector to replace the whole pCMV promoter. New plasmid was called phTERT-pEGFP-N1. An enhanced phTERT promoter named phTERTe promoter was also synthesized, which included the enhancer of pCMV and phTERT promoter. Other plasmids were constructed in the same way, such as phTERTe-pEGFP-N1, phTERTe-pcDNA4-ADI-cmyc, phTERTe-pcDNA4-p53-cmyc, phTERTe-Ad5-ADI-cmyc, phTERTe-Ad5-p53-cmyc, and so on. Thereinto, pcDNA^TM^4/TO/myc-His vector and pacAd5/CMV/K-N/pA vector were used for constructing above plasmids. Table 1 displayed all of the information about the plasmid construction including restriction enzymes and primers.

The shuttle plasmids of recombinant Ad5 were co-transfected into 293A cells with adenovirus backbone plasmid pacAd5 from Cell Biolabs INC. The recombinant adenoviruses were packaged out by homologous recombination in the cell. Purification of the virus was performed using the CsCl gradient centrifugation. Titers of recombinant virus were determined by TCID50 (Tissue culture infective dose).

### Quantitative PCR analysis for the endogenous hTERT

The expression of endogenous hTERT protein was assayed by quantitative PCR (qPCR). Briefly, total RNA for each cell line was extracted using TRIzol (Life Technologies) according to the manufacturer's instructions for monolayer cells in a 6-well plate format. Reverse transcription was performed from 3 μg total RNA using oligo(dT) and RevertAid Reverse Transcriptase (Thermo Scientific) according to the supplier's instructions. Quantitative PCR was performed with SuperReal PreMix SYBR Green (TIANGEN) using an Applied Biosystems 7500 Fast Real-Time PCR System (Life Technologies). 2^-ΔΔCt^ in relative quantification analysis method was used to calculate the change fold of hTERT mRNA among the different cell lines. PCR primers included (5′ to 3′) hTERT_sense_ (CGTACAGGTTTCACGCATGTG) and hTERT_antisense_ (ATGACGCGCAGGAAAAATG).

### phTERT promoter activity assays

The resulting GFP reporter vectors, phTERT-pEGFP-N1 and phTERTe-pEGFP-N1, were transfected with lipo2000 (invitrogen) into individual tumor cell lines followed by GFP analysis. Briefly, GFP activity Detection by fluorescence microscope was normalized to the activity of phTERT promoter after 24-28h. Western blot analysis was processed to verify the expression level of GFP protein at the same time.

### Western blot analysis

Five micrograms of protein were electrophoresed in 10% SDS-PAGE gels and blotted to polyvinylidine difluoride membranes. Specific primary antibodies (ProteinTech Group Inc.) were detected with peroxidase-labeled secondary antibodies (Amersham, Piscataway, NJ) using SuperSignal West Dura Extended Duration Substrate (Pierce Chemical) per manufacturer's instructions.

### Determination of apoptosis by flow cytometry

The extent of apoptosis was determined by the flow cytometric measurement through Annexin V-FITC apoptosis detection kit (Beyotime, China). Cells were divided into untreated, p53-adnovirus--treated, and ADI-adenovirus-treated groups. After 48 h, cells were harvested and washed twice with PBS. Then, cells were stained in 1 mL Annexin V binding buffer with 10 μL of PI solution and 5 μL of Annexin V-FITC for 10 min at room temperature and analyzed by flow cytometry.

### Animal model in mice and tumor dissolution experiments

For the intratumoral injection model, 5 ×10^6^ of BEL7402 cells were inoculated into the hind flank of 4-week-old male Kunming mice. BEL7402 cells were digested from the culture plates by the trypsin, and then diluted into 3×10^6^/ml after trypan blue staining and microscopic count. 0.2 ml of cells was injected into the right subcutaneous axillary of each mouse. The soya-bean size tumors grew up after 1-3 weeks. What's more, the tumors didn't subside during the rest life of mice. So the heap-tumor model was established successfully.

When palpable tumors developed into almost 50 mm^3^, mice were randomly divided into three groups. Each group had 6 mice and was injected with one kind of adenovirus, such as ADI-adenovirus, p53-adenovirus and blank adenovirus. Adenovirus titer was l×10^9^ pfu/ml. 100ul virus was injected into tumors every two days. Tumor's sizes and mice weights were measured at the same time.

### Immunohistochemistry

Tumors were taken away from mice and made into paraffin sections. Sections were treated as the usual procedures. After deparaffinized, dehydrated, inactivated and blocked, sections were processed with hematoxylin-eosin staining (HE staining) firstly. Hematoxylin was used to show the nuclear morphology. Eosin was used to stain cytoplasm. Then, immunohistochemistry staining was carried out with anti-MYC tag (mouse monoclonal antibody IgG1a, CA: BM0238, BOSTER LTD, Wuhan, China.) (dilution 1:100) to detect the expression of exogenous proteins transferred by adenovirus. Diaminobenzidine tetrahydrochloride (DAB) was developed to show the yellow color. Hematoxylin was also utilized to stain cellular nucleus.
